# Nuclear Overhauser Enhancement Imaging of Glioblastoma at 7 Tesla: Region Specific Correlation with Apparent Diffusion Coefficient and Histology

**DOI:** 10.1371/journal.pone.0121220

**Published:** 2015-03-19

**Authors:** Daniel Paech, Sina Burth, Johannes Windschuh, Jan-Eric Meissner, Moritz Zaiss, Oliver Eidel, Philipp Kickingereder, Martha Nowosielski, Benedikt Wiestler, Felix Sahm, Ralf Omar Floca, Jan-Oliver Neumann, Wolfgang Wick, Sabine Heiland, Martin Bendszus, Heinz-Peter Schlemmer, Mark Edward Ladd, Peter Bachert, Alexander Radbruch

**Affiliations:** 1 Department of Neuroradiology, University of Heidelberg Medical Center, Heidelberg, Germany; 2 Neurooncologic Imaging, Department of Radiology, Deutsches Krebsforschungszentrum (DKFZ), Heidelberg, Germany; 3 Department of Medical Physics in Radiology, Deutsches Krebsforschungszentrum (DKFZ), Heidelberg, Germany; 4 Department of Neurology, Innsbruck Medical University, Innsbruck, Austria; 5 Department of Neurology, University of Heidelberg Medical Center, Heidelberg, Germany; 6 Department of Neuropathology, University of Heidelberg Medical Center, Heidelberg, Germany; 7 Department of Radiology, Deutsches Krebsforschungszentrum (DKFZ), Heidelberg, Germany; 8 Department of Neurosurgery, University of Heidelberg Medical Center, Heidelberg, Germany; Julius-Maximilians-Universität Würzburg, GERMANY

## Abstract

**Objective:**

To explore the correlation between Nuclear Overhauser Enhancement (NOE)-mediated signals and tumor cellularity in glioblastoma utilizing the apparent diffusion coefficient (ADC) and cell density from histologic specimens. NOE is one type of chemical exchange saturation transfer (CEST) that originates from mobile macromolecules such as proteins and might be associated with tumor cellularity via altered protein synthesis in proliferating cells.

**Patients and Methods:**

For 15 patients with newly diagnosed glioblastoma, NOE-mediated CEST-contrast was acquired at 7 Tesla (asymmetric magnetization transfer ratio (MTR_asym_) at 3.3ppm, B1 = 0.7 μT). Contrast enhanced T1 (CE-T1), T2 and diffusion-weighted MRI (DWI) were acquired at 3 Tesla and coregistered. The T2 edema and the CE-T1 tumor were segmented. ADC and MTR_asym_ values within both regions of interest were correlated voxelwise yielding the correlation coefficient r_Spearman_ (r_Sp_). In three patients who underwent stereotactic biopsy, cell density of 12 specimens per patient was correlated with corresponding MTR_asym_ and ADC values of the biopsy site.

**Results:**

Eight of 15 patients showed a weak or moderate positive correlation of MTR_asym_ and ADC within the T2 edema (0.16≤r_Sp_≤0.53, p<0.05). Seven correlations were statistically insignificant (p>0.05, n = 4) or yielded r_Sp_≈0 (p<0.05, n = 3). No trend towards a correlation between MTR_asym_ and ADC was found in CE-T1 tumor (-0.31<r_Sp_<0.28, p<0.05, n = 9; p>0.05, n = 6). The biopsy-analysis within CE-T1 tumor revealed a strong positive correlation between tumor cellularity and MTR_asym_ values in two of the three patients (r_Sp_^patient3^ = 0.69 and r_Sp_^patient15^ = 0.87, p<0.05), while the correlation of ADC and cellularity was heterogeneous (r_Sp_^patient3^ = 0.545 (p = 0.067), r_Sp_^patient4^ = -0.021 (p = 0.948), r_Sp_^patient15^ = -0.755 (p = 0.005)).

**Discussion:**

NOE-imaging is a new contrast promising insight into pathophysiologic processes in glioblastoma regarding cell density and protein content, setting itself apart from DWI. Future studies might be based on the assumption that NOE-mediated CEST visualizes cellularity more accurately than ADC, especially in the CE-T1 tumor region.

## Introduction

As the diagnostic gold standard in neurooncology [1], MRI is supposed to provide information on tumor grade, tumor progression, response to therapy and biopsy planning sites. When developing new MRI contrasts, it is important not only to show differences to basic contrasts such as T1 and T2 weighted images—it should also complement and provide additional information to already established functional MRI imaging sequences such as diffusion-weighted MRI (DWI), perfusion-weighted MRI and susceptibility-weighted MRI (SWI).

Nuclear Overhauser enhancement (NOE)-mediated imaging is a new MRI contrast and is one type of Chemical Exchange Saturation Transfer (CEST) imaging [2]. Within the field of neuroradiology, CEST imaging has been proved useful for the evaluation of brain tumors [2–5] and ischemia [6, 7]. CEST is based on the saturation transfer between exchanging protons of tissue proteins and bulk water [8]. CEST data are commonly acquired using asymmetry analysis (MTR_asym_(Δω) = Z(-Δω)- Z(Δω)) with respect to the water frequency set at Δω = 0 and normalized to unsaturated signal (S_0_) creating a Z-spectrum.

The most commonly used CEST-contrast is the Amide Proton Transfer (APT). The APT contrast arises from –NH groups of mobile proteins and peptides and occurs at 3.5 ppm in the Z-spectrum [4]. It shows an increased signal in brain tumors [3, 4] and correlates with cell density in gliomas [9]. It was also reported that this method may differentiate tumor recurrence from radiation necrosis after radiation therapy [10].

NOE mediated effects on the other hand occur due to dipolar spin-spin interaction of immobile protons with exchanging protons within macromolecules; they are also called exchange-relayed NOEs [11]. The NOE-based signals originate from mobile macromolecular components such as proteins that are composed of aliphatic and olefinic molecules with a spectral range upfield from the water frequency at -2 to-5 ppm [2]. Accordingly, the NOE is reported to be influenced by protein content, protein folding and protein mobility within tissue [12].

Our approach was to investigate a possible link between cellularity and the NOE-contrast in tumors which is based on the assumption that protein metabolism is a crucial factor for glioblastoma cells. It is known that significant metabolic reprogramming occurs in astrocytes as they turn malignant, for example the abundance of enzymes responsible for protein synthesis, processing and degradation varies in comparison to normal astrocytes [13, 14]. Consequently, the expressed proteins in glioblastoma cells differ significantly from those in normal astrocytes [15] which might influence the NOE-signal in areas of condensed tumor cells. A histopathological correlate to the NOE-effects in glioblastoma has not been reported yet. One key to determining the clinical value of this new contrast is to test its ability to demarcate areas of malignancy or infiltrative tumor growth for planning of biopsy, resection margins or radiation therapy. We investigated a possible association of NOE-effects and tumor cellularity with two coherent approaches.

Firstly, we analyzed the NOE mediated CEST in comparison to an established MRI contrast, the apparent diffusion coefficient (ADC), derived from diffusion weighted MRI. This approach is based on the findings of numerous studies that showed inverse correlation between ADC and tumor cellularity [16–19]. Besides, low ADC values indicate a worse prognosis in glioblastoma patients [20, 21].

Typical region specific signal intensity gradations can be observed for both contrasts in glioblastoma: The ADC value generally increases towards tumorous tissue and necrosis compared to peritumoral edema [22], whereas NOE mediated CEST effects turned out to decrease from normal appearing white matter via peritumoral edema to CE-T1 tumor and necrosis [2, 12, 23]. Since the NOE is located upfield from the water resonance, reduced NOE effects display as hyperintensities on the MTR_asym_ contrast.

The second approach was to correlate MTR_asym_ values with tumor cellularity obtained from stereotactic biopsies. To the best of our knowledge, such an analysis has not been conducted before. In our patient collective, biopsy data was obtained from three patients and our methodic approach promises an interesting first insight into how MTR_asym_ correlates with underlying histopathologic findings in glioblastoma, primarily with tumor cellularity.

## Patients and Methods

### Patients

Fifteen patients (4 female, 11 male; age: 61.6 ± 14.1 years) with newly diagnosed and subsequently histopathologically confirmed glioblastoma were included in this prospective study. The study was approved by the Medical Ethics Committee of the University of Heidelberg and written informed consent was received from all participants before enrollment.

### Conventional MRI at 3T

All images were acquired on a 3T whole body MR imaging system (MagnetomVerio/ Trio TIM; Siemens Healthcare, Erlangen, Germany) with a 12-channel head matrix coil. CE-T1 weighted images (TE = 4.04 ms, TR = 1710 ms, FoV 256 x 256 mm^2^, resolution 512 x 512, slice thickness 1 mm) and T2-weighted images (TE = 89 ms, TR = 5140 ms, FoV 172 x 229 mm^2^, resolution 384 x 230, slice thickness 4 mm) were performed. SWI data were obtained with a three-dimensional fully flow-compensated gradient-echo sequence (27/19.7, flip angle 15°, section thickness 2.5 mm).

### Diffusion imaging at 3T

The DWI was performed for all patients as part of the routine tumor brain protocol on a 3T whole-body MR tomograph (Magnetom Verio/ TrioTIM; Siemens Healthcare, Erlangen, Germany). The DWI images were obtained using parallel imaging (GRAPPA) and an echo planar read-out with the following parameters: TR/TE 5300/90 ms, b = 0 mm^2^/s and b = 1200 mm^2^/s, 3 directions, FoV 230x 230 mm^2^, matrix 130x130, slice thickness 5 mm. ADC maps were calculated with a commercially available software (syngo MR; Siemens Healthcare).

### CEST-MRI at 7T

1–5 days after the 3T MRI, the NOE mediated CEST sequence was obtained with a 7T whole body MRI scanner (Magnetom 7T; Siemens Healthcare, Erlangen, Germany) using a 24 channel head coil and a centric-reordered three-dimensional gradient echo sequence [24] with the same parameters that were employed in our institution by Paech et al [23] before: TR/TE 12/2.88 ms, FoV phase = 78.125%, matrix 128x100, 26 slices, resolution = 1.8 mm x 1.8 mm x 2 mm, BW = 320 HZ/px, FA = 10°, GRAPPA acceleration factor 3. The pulse train before each segment of the 3D stack consisted of 5 Gaussian pulses with a duration of 100 ms per pulse and a pulse-train-average amplitude of B_1_ = 0.7 μT.

An interpulse delay of 100 ms was set because of SAR limitations. Thus, the effective saturation time was 900 ms. Thirteen equidistant frequency offsets between -4 and +4 ppm and the additional M_0_ image were acquired, resulting in an acquisition time of 9 min 30 s. Employing a cubic spline interpolation, the minima of the Z-spectra were determined resulting in a B_0_ deviation map, which is used to correct B_0_ inhomogeneities. For each pixel, the reduced water magnetization M was normalized by the unsaturated magnetization M_0_ yielding Z = M/M_0_. Z plotted as a function of the irradiation frequency offset Δω formed the Z-spectrum. The relative contributions of different CEST-effects can be tuned by varying saturation pulse length (t_sat_) and field strength (B_1_) [25]. Zhou et al [25] found that the MTR_asym_(3.5 ppm) contrast at 0.6 μT is predominantly contributed by the NOE and Jin et al [26] state an NOE-dominated contrast for B_1_ = 1μT. APT-mediated effects that occur at 3.5 ppm [4] can confound the measurement of exchange relayed NOE occuring in the whole range from -2 to-5 ppm [2, 27]. Therefore, the CEST-signal intensity was defined as magnetization transfer ratio asymmetry (MTR_asym_) at 3.3 ppm and B_1_ = 0.7 μT where CEST-effects are mainly NOE-dominated.

MTR_asym_ was calculated as:

$$MTRasym\left(3.3\;ppm\right)\;=\;Z\left(\Delta \omega \;=\;-3.3\;ppm\right)—Z(\Delta \omega \;=\;+3.3\;ppm)$$

Consequently, high MTR_asym_ values mark decreased NOE-mediated CEST-effects. The CEST-contrast was windowed between MTR_asym_ values from -10% to 5%. Images of all sequences (CE-T1, T2, MTR_asym_, DWI, SWI) were co-registered with the software MITK [28]. The registration of all data types was done by an automatic multi modal rigid registration algorithm [29] and the registration results were validated by clinical experts before being used in the analysis process.

### Volumetric segmentation

**Fig 1:**
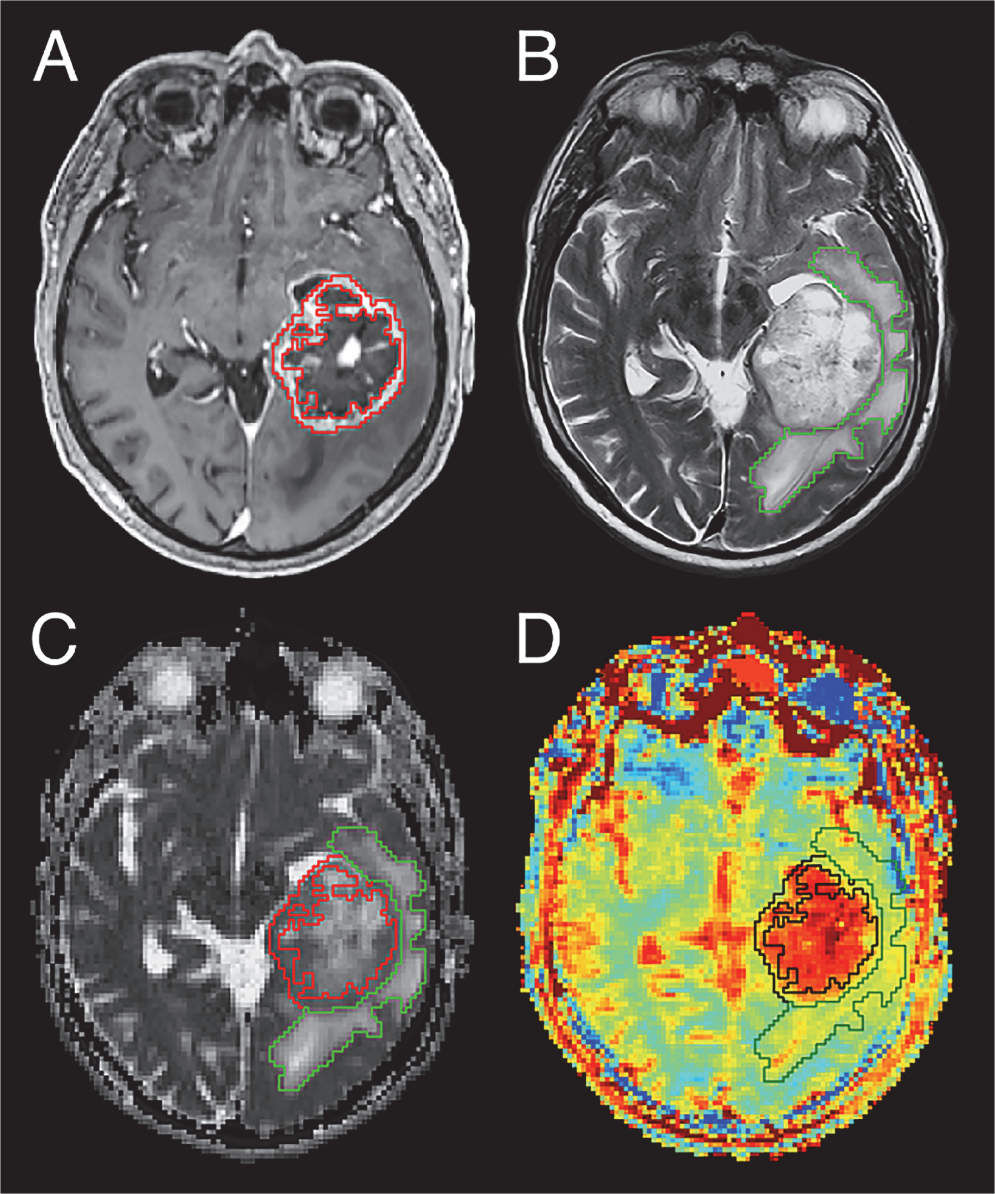
Segmentation of the regions of interest (ROI) CE-T1 tumor and T2 peritumoral edema. Glioblastoma of the left temporal lobe in patient 6. Coregistered images from the different sequences showing an exemplary slice of the whole tumor volume for this patient. **A)** CE-T1 tumor ROI (red line) segmented on CE T1-weighted image while thoroughly excluding central necrosis. **B)** T2 peritumoral edema ROI (green line) segmented on T2-weighted image. **C)** Coregistered ADC map and **D)** MTR_asym_ contrast illustrating both ROIs. The glioblastoma tumor and the cerebrospinal fluid in sulci and ventricles display hyperintense on NOE mediated CEST based on MTR_asym_.

Data analysis was done on MITK [28]. Two types of regions of interest (ROIs) that depicted the lesion were manually segmented as shown for patient 6 (Fig. 1). On the CE-T1 image, the contrast enhancing area was delineated (CE-T1 tumor), excluding necrotic parts thoroughly. On the T2 image, the area of peritumoral edema was selected, again excluding areas of contrast enhancement and necrosis. The segmentation was done in all slices, using a volumetric approach. To avoid decreased ADC values caused by the confounding factor of micro-bleeds within the glioblastoma, we also used coregistrated SWI sequences to eliminate areas of SWI hypointensity in both ROIs [30–32]. For further statistical analysis, a voxel-wise read-out of MTR_asym_ values and registered corresponding ADC values was performed.

### Stereotactic biopsy and calculation of tumor cellularity

Histologic specimens were obtained from Patient 3, 4 and 15 via stereotactic biopsies which were performed for histopathologic confirmation of the diagnosis instead of direct surgical resection. A stereotactic biopsy ring was mounted to the patient’s skull. The attending Neurosurgeon (JON) calculated a trajectory on the intraoperative CE-T1 MRI (iPS Software, inomed Medizintechnik GmbH, Emmendingen, Germany) from an entry point at the skull to a target point in the contrast enhancing zone of the CE-T1 image. The coordinates of those points were transferred to the intraoperative CE-T1 image, the MTR_asym_ image and the ADC image using a custom in-house MATLAB script (MATLAB 2014b, The Mathworks, Natick, MA, USA) after having coregistrated all sequences on the intraoperative CE-T1 with an automatic multi modal rigid registration algorithm in MITK [28, 29]. A total of 12 biopsies were taken along the trajectory of every patient and the distance from the target point in mm was noted in the pathology report. For each position of a biopsy, the average MTR_asym_ and average ADC of the surrounding 3x3x3 voxels (27 mm^3^) were obtained in order to correct for possible spatial inaccuracy.

**Fig 2:**
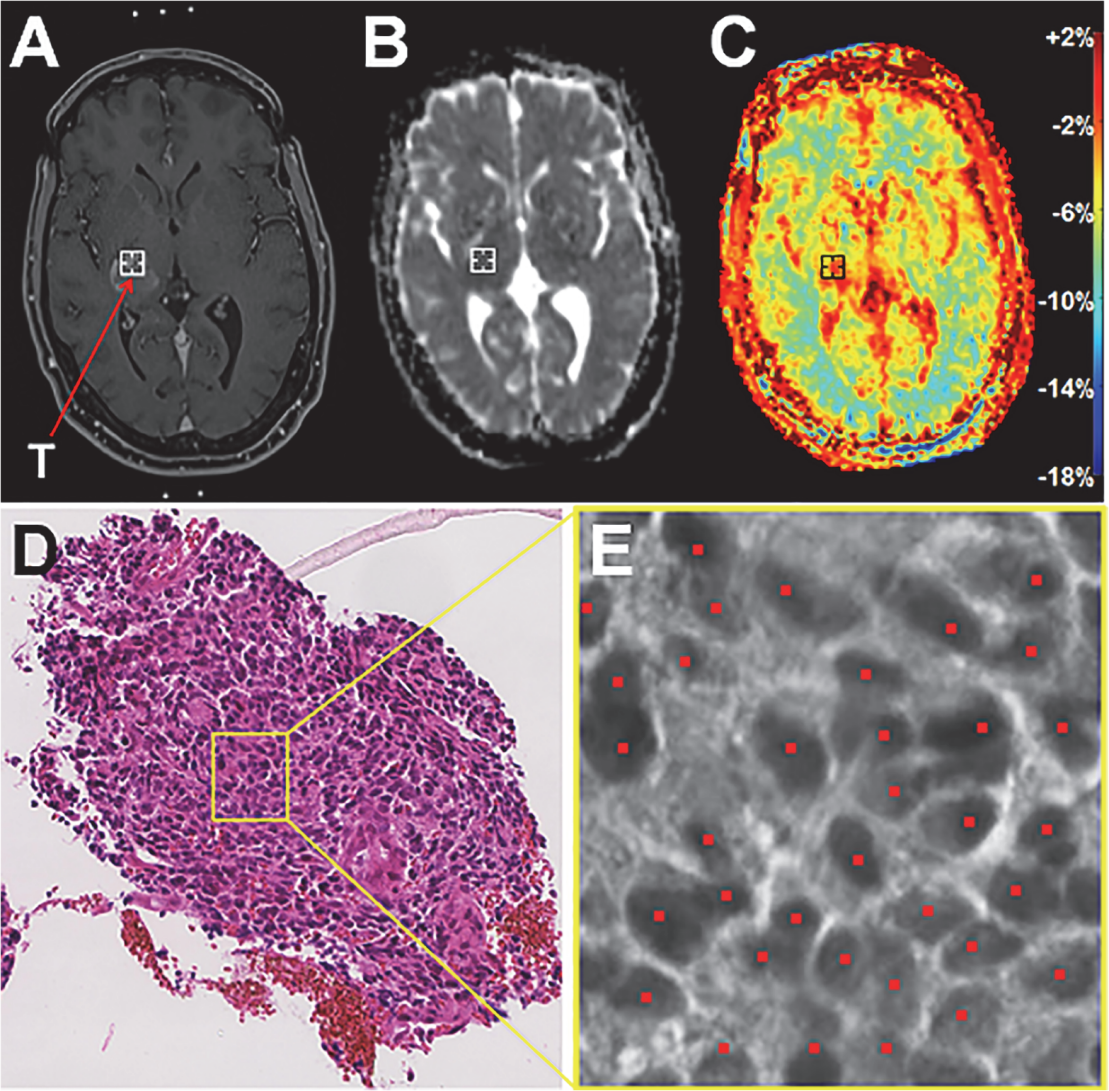
Correlation of the biopsy point on the MTRasym image and the ADC image with histology and semi-automatic cell counting. **A)** Target point T of the biopsy trajectory on the intraoperative CE-T1 image of a 45-year-old male with glioblastoma (patient 15). It lies in the contrast-enhancing area. Marks of the stereotactic biopsy ring are visible frontally and occipitally. **B)** Target point T on the ADC image which was coregistered to the intraoperative CE-T1 image in MITK with an automatic multi modal rigid registration algorithm. The average ADC value of 3x3x3 voxels (= 27mm^3^) was read out to account for possible inaccuracies in coregistration and biopsy sampling. In the shown example, it yielded ADC = 659mm^2^/s. **C)** Target point T on the MTR_asym_ image which was coregistered to the intraoperative CE-T1 image in MITK with an automatic multi modal rigid registration algorithm. The average MTR_asym_ value of 3x3x3 voxels (= 27mm^3^) was read out to account for possible inaccuracies in coregistration and biopsy sampling. In the shown example, it yielded MTR_asym_ = -1,72% **D)** Corresponding slice of the 1 mm^3^ biopsy specimen (HE stain) in x20 magnification obtained at the target point T. **E)** Exemplary section to illustrate semi-automatic cell counting with the ImageJ plugin ITCN. Tumor cells that were recognized by the algorithm are marked red. Overall cell density of the shown biopsy specimen was 1684 cells/mm^2^.

All biopsies were scanned at x20 magnification and analyzed by a Neuropathologist. To calculate cell density, biopsy images were postprocessed using NIH ImageJ, 64-bit version [33]. Cell density was calculated semi-automatically with the ImageJ plugin ITCN [34], which required an estimate of cell width and cell spacing as input. A Neuropathologist (FS) checked the correctness of cell detection and cell count. The procedure of correlating the position of the biopsy on the MRI images with the results of histopathologic cell counting is illustrated in Fig. 2.

### Statistical Analysis

#### Correlation analysis of MTR_asym_ and ADC values

The obtained data from the region specific voxelwise readout was used for statistical evaluation with SigmaPlot version 12.5 (SystatSoftware, Inc., San Jose California USA). For each patient, a Spearman correlation analysis was performed for MTR_asym_ values and corresponding ADC values in the two ROIs. Furthermore, 95% confidence intervals were conducted employing the approach of Bonett and Wright [35].

We interpreted the Spearman correlation coefficient r_Sp_ as suggested by Zou et al [36], where r_Sp_≈0 means no association, r_Sp_≈±0.2 is a weak correlation and r_Sp_≈±0.5 is a moderate correlation.

#### Correlation analysis of MRI data and tumor cellularity

We obtained corresponding MTR_asym_ values, ADC values and cell density values for 12 biopsy sites per patient as described above. In a first step, Spearman correlation was performed with SPSS (IBM SPSS Statistics 21). Scatterplots were generated and a linear regression model was fitted if possible. For all statistical analyses the level of significance was set at p<0.05.

## Results

### Correlation between MTR_asym_ and ADC in the CE-T1 tumor region

Of the 15 patient-individual correlation analysis for the CE-T1 tumor ROI, two patients tended towards a weak positive correlation between ADC and MTR_asym_ (r_Sp_ = 0.19 and 0.28, p<0.001) and two patients showed a weak negative correlation (r_Sp_ = -0.17 and -0.31, p<0.05 and <0.001). In the other 11 patients, the correlation was either not significant (p>0.05, n = 6) or r_Sp_ was too small to claim any association between ADC and MTR_asym_ (n = 5). Consequently, there is no tendency towards any correlation between ADC and MTR_asym_ values in the area of CE-T1 tumor in glioblastoma. The results of the correlation analysis between ADC and MTR_asym_ are summarized in Table 1.

**Table 1:** Region specific Spearman correlation analysis of MTR_asym_ and ADC contrast.

Patient	r_Sp (CE-T1)_	p-value	total voxel	r_Sp (T2 edema)_	p-value	total voxel
**1**	−0.07	>0.05	658	0.09*	<0.001	2577
**2**	−0.17	<0.05	316	0.16*	<0.001	1083
**3**	0.02	>0.05	325	0.18*	<0.05	198
**4**	0.06	>0.05	98	0.20*	<0.001	851
**5**	0.04	>0.05	242	0.20*	<0.001	1077
**6**	0.00	>0.05	1515	0.23*	<0.001	3044
**7**	0.19*	<0.001	1931	0.26*	<0.001	1254
**8**	−0.1	<0.05	663	0.29*	<0.001	3759
**9**	0.28*	<0,001	713	0.53*	<0.001	2366
**10**	0.12*	<0.001	1662	−0.07	<0.001	3261
**11**	−0.31	<0.001	1967	−0.13	<0.05	414
**12**	0.07*	<0.05	916	0.03	>0.05	1269
**13**	0.07*	<0.05	2172	0.03	>0.05	1203
**14**	0.13*	<0.001	1720	−0.01	>0.05	1080
**15**	−0.01	>0.05	225	−0.04	>0.05	170

*significant positive correlations. Correlation coefficients (r_Sp_) sorted by their values for the ROI T2 peritumoral edema. Insignificant values (p >0.05) or values around r_Sp_≈0 [-0.15:+0.15] are not considered as tendency towards any correlation.

### Correlation between MTR_asym_ and ADC in the T2 peritumoral edema region

For the T2 peritumoral edema ROI, we found a trend towards a weak positive correlation between ADC and MTR_asym_ as seven of the 15 patients classify as weakly correlating (r_Sp_ = 0.16; 0.18; 0.20; 0.20; 0.23; 0.26; 0.29) and one as moderately correlating (r_Sp_ = 0.53, p<0.001). In the other seven patients, the correlation was either not significant (p>0.05, n = 4) or r_Sp_ was too small to claim any association between ADC and MTR_asym_ (n = 3) (results are summarized in Table 1).

**Fig 3:**
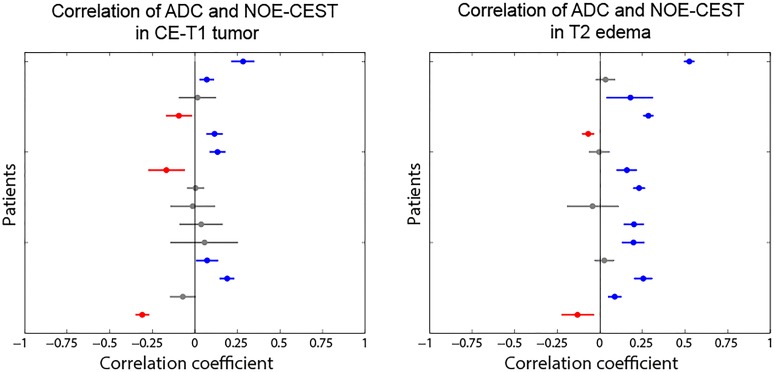
Patient-individual Spearman correlation coefficients (r_Sp_) with 95% confidence intervals. Positive correlations are marked blue, negative correlations are red, insignificant correlations (p>0.05) are grey. No trend towards any correlation between MTR_asym_ and ADC could be found in the CE-T1 tumor region (left diagram), since correlation coefficients scatter around r_Sp_≈0. For the T2 edema region (right diagram) a trend towards a positive correlation could be observed. Eight of fifteen patients correlate weakly or moderately positive, while seven coefficients are insignificant (n = 4) or too low to claim an association (n = 3). The trend towards a positive correlation within the T2 edema region is suspected to be due its more homogeneous structure, compared to the CE-T1 tumor which is characterized by different cell types, vasculogenesis and necrotic foci.

**Fig 4:**
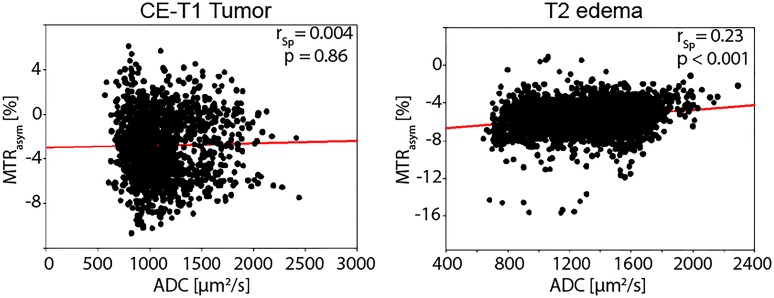
Voxelwise correlation of ADC and MTR_asym_ contrast for patient 6. The shown scatterplots correspond to the volumetric segmentation of the CE-T1 tumor and T2 peritumoral edema region for an exemplary patient (Patient 6). A linear regression (red line) was additionally plotted for both regions. For the CE-T1 tumor, no correlation could be observed over the 1515 voxel, whereas a weak positive correlation over the 3044 voxel of the T2 edema region of this patient is found. The weakly positive correlation within the T2 edema signifies that high MTR_asym_ values (caused by decreased NOE effects) tend to correspond to high ADC values in this region. The graphs represent the generally observed trend within the patient collective.

Fig. 3 illustrates all r_Sp_-values with their 95% confidence intervals for both ROIs. The scatterplot in Fig. 4 is a representative example to illustrate the raw data obtained from the voxelwise readout as this patient shows no significant correlation in the area of CE-T1 tumor and a weak positive correlation within the T2 peritumoral edema.

### Correlation analysis between MRI data and cell density from stereotactic biopsy specimens in the CE-T1 tumor region

**Fig 5:**
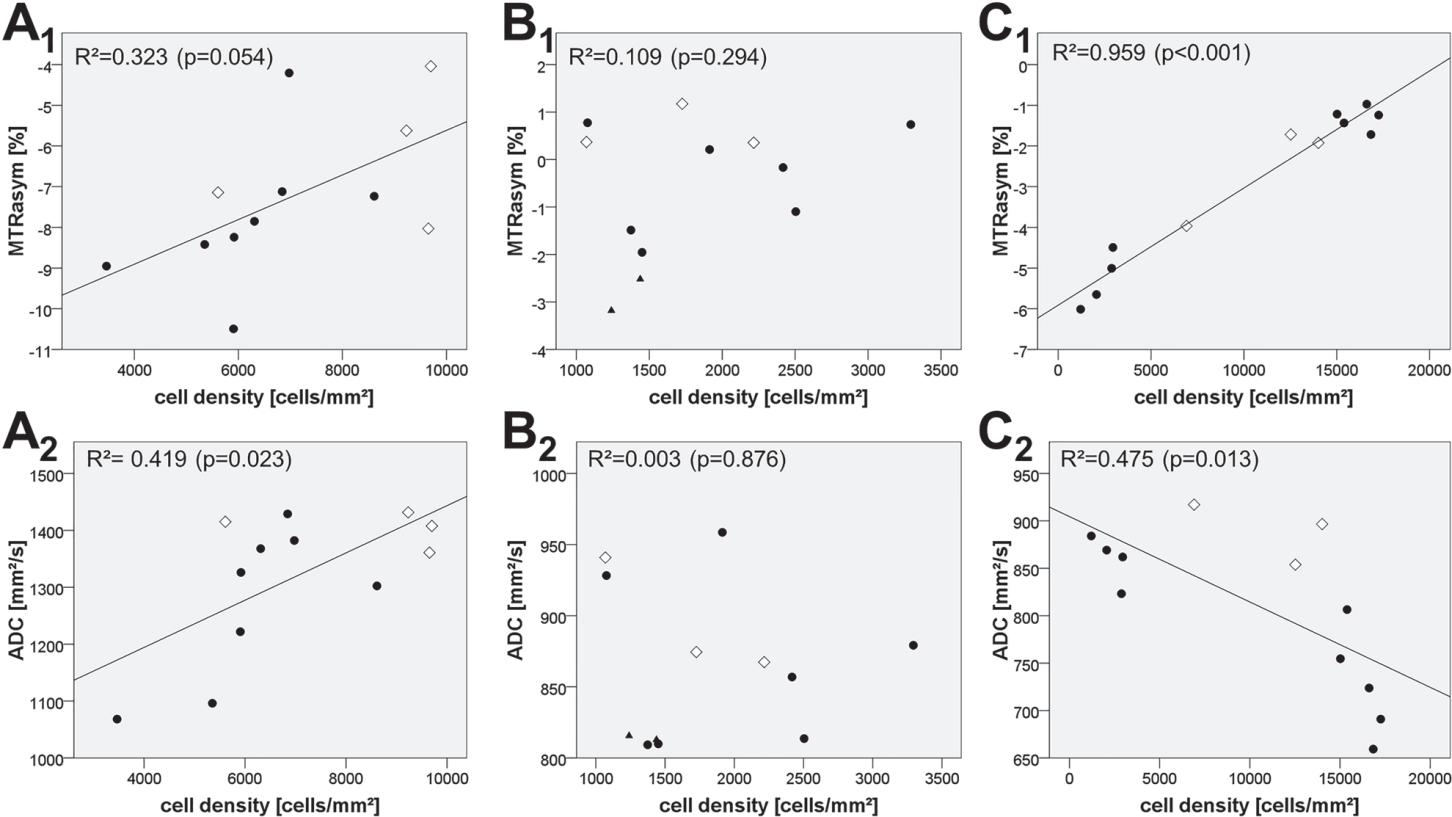
Tumor cell density and the corresponding MTR_asym_ values/ADC values at the origin of the biopsy. • Biopsy from contrast-enhancing parts on CE-T1. ◊ Biopsy from necrotic areas as visible on CE-T1. ▲Biopsy from non-enhancing areas on CE-T1 (“edema”). **A_1_/A_2_)** 12 biopsies were obtained along a trajectory in a 67-year-old female with glioblastoma (patient 3). Linear regression of cell density and MTR_asym_ values yielded R^2^ = 0.323 (p = 0.054). Linear regression of cell density and ADC values yielded R^2^ = 0.419 (p = 0.023). **B_1_/B_2_)** 12 biopsies were obtained along a trajectory in a 60-year-old female with glioblastoma (patient 4). Linear regression of cell density and MTR_asym_ values yielded R^2^ = 0.109 (p = 0.294). Linear regression of cell density and ADC values yielded R^2^ = 0.003 (p = 0.876). **C_1_/C_2_)** 12 biopsies were obtained along a trajectory in a 45-year-old male with glioblastoma (patient 15). Linear regression of cell density and MTR_asym_ values yielded R^2^ = 0.959 (p<0.001). Linear regression of cell density and ADC values yielded R^2^ = 0.475 (p = 0.013).

The correlation analysis of the MTR_asym_ values and the biopsy-derived cell densities yielded strong positive correlations for two of the three patients (r_Sp_^patient3^ = 0.685 and r_Sp_^patient15^ = 0.867, p<0.05). In patient 4, the correlation coefficient was also positive but not statistically significant (r_Sp_^patient4^ = 0.126, p = 0.697) (Table 2). The maximum values for the obtained cell densities were 9699 cells/mm^3^ (Patient 3) and 17256 cells/mm^3^ (Patient 15) from the two significantly positive correlating analyses, while the maximum cell density in the insignificantly correlating analysis was 3293 cells/mm^3^ (Patient 4). The correlation of ADC values with the corresponding histologic cell densities resulted in significantly negative (r_Sp_^patient15^ = -0.755, p = 0.005) and insignificant (r_Sp_^patient4^ = -0.021, p = 0.948; r_Sp_^patient3^ = 0.545, p = 0.067) Spearman rank correlation coefficients (Table 2). Consequently, there was no trend towards an overall correlation for ADC and cell density in CE-T1 tumor. Scatterplots and linear regression models are shown in Fig. 5.

**Table 2:** Correlation analysis of MTR_asym_ values and ADC values with the cell density of corresponding biopsy sites.

Patient ID	r_Sp_ of MTR_asym_ and cell density	r_Sp_ of ADC and cell density	number of biopsies	maximum cell density [cells/mm^3^]
**Patient 3**	0.685* (p = 0.014)	0.545 (p = 0.067)	12	9699
**Patient 4**	0.126 (p = 0.697)	−0.021 (p = 0.948)	12	3293
**Patient 15**	0.867* (p = <0.001)	−0.755* (p = 0.005)	12	17256

* significant correlations are marked. r_Sp_ = Spearman rho

## Discussion

In this study, we investigated the properties of the NOE-mediated CEST-signal in a region-specific approach regarding tumor cellularity, in which we used the ADC and histologic specimens from stereotactic biopsies as matters of comparison.

### CE-T1 tumor region

As a principle finding of this study, we showed that in the area of CE-T1 tumor, ADC and MTR_asym_ did not correlate suggesting that these MRI contrasts have distinct underlying principles and that the NOE-mediated CEST contrast provides information about glioblastoma that is different from the one obtained in DWI.

Furthermore, the correlation analysis between MTR_asym_ and cell density, obtained from biopsy specimens, revealed a strong positive correlation in two cases while one correlation coefficient was positive but statistically not significant. Remarkably, the maximum cell density from the patients in which we found a strong positive correlation is much higher than in the patient without significant correlation (17256 and 9699 cells/mm^3^ versus 3293 cells/mm^3^). This might lead to the suggestion that the obtained correlation between MTR_asym_ and cell density can only be observed in tumors of high cell densities.

The analogous correlation analysis between ADC and cell density produced very variable results including both significant negative and insignificant correlation coefficients (r_Sp_^patient3^ = 0.545, p = 0.067); r_Sp_^patient4^ = -0.021, p = 0.948; r_Sp_^patient15^ = -0.755, p = 0.005), questioning whether the ADC is a suitable marker for cellularity in the CE-T1 tumor zone. In this regard, the heterogeneity of our correlation analysis is in agreement with inconsistent results that are found in literature concerning an association of ADC and cellularity:

Chen et al [37] reported a pooled correlation coefficient of ρ = -0.61 for ADC and cellularity whereas Stadlbauer et al [38] found a positive correlation (ρ = 0.41) in an analysis of 77 biopsies.

Moreover, our results are in accordance with Xu et al [39], as their MTR*_asym_ signal in rat 9L glioma tumors at 9.4 Tesla and B1 = 1μT (defined as Z(-3.5ppm)-Z(3.5ppm)) did correlate with ADC, while the more NOE-specific signal NOE* (based on asymmetry analysis at ±3.5 ppm with a three-offset method) did not correlate with ADC.

While one cannot deduct any certain conclusions from such a small number of patients, it is still interesting to look at possible hypotheses that might explain a positive correlation of MTR_asym_ and cellularity. Concerning the MTR_asym_ values, one has to bear in mind that low MTR_asym_ values represent high NOE-mediated exchange rates (which is represented by a drop in the Z-spectrum at -3.3ppm) and vice versa. This would mean a decreasing NOE signal with increasing cellularity. Possible factors influencing the NOE signals are protein concentration, folding and mobility.

Regarding protein content, a lowered concentration would be a possible explanation for decreased NOE effects. In accordance with this hypothesis, it has been suggested that there is a high water content (or decreased protein content per voxel, respectively) in glioblastoma tumor and necrosis [2, 12] as it has long been known that the water content is increased in tumors of all entities [40]. However, a lowered protein concentration in hypercellular regions seems counterintuitive (even though high cellularity does not necessarily imply high protein content). There are recent investigations that report an increased protein concentration within glioblastoma tumor and necrosis [41] which would conflict this thesis. Xu et al [39] also found that the rat 9L glioma tumors had slightly, but not significantly higher protein concentrations. As it remains inconclusive how protein concentration and NOE signals are linked in glioblastoma, it has also been hypothesized that protein unfolding or misfolding might be responsible for a decreased NOE signal. As a tumor cell is exposed to a higher level of stress (nutrition supply, proliferation), misfolding and unfolding of proteins will occur [42]. This triggers an unfolding protein response with mechanisms to resolve the protein-folding defects or to guide the cell into apoptosis and an upregulation of these mechanisms is known to serve cancer cells as a survival strategy [42]. Again, whether an increased incidence of unfolded or misfolded proteins in areas of high cellularity is responsible for a drop in the NOE-signal cannot be confirmed by our experiment, but it remains an interesting hypothesis.

Finally, altered protein mobility might result in changes in NOE effects: In glioblastoma, a disruption of cell structures might increase the mobility of proteins and thus decrease the exchange relayed NOE signals due to the greater distances between exchanging protons.

Ultimately, an influence of the pH on the NOE-signal has to be considered as other studies have shown a small increase of the NOE-effects with pH (approximately 0.6% per pH) [2, 26]. Extracellular pH is known to decrease in tumor cells from 7.4 in healthy tissue down to 6.7 in anoxic tumor regions [43]. Consequently, through regulation pathways, the pH increases in the intracellular compartment of brain tumors (pH = 7.3) compared to normal brain tissue (pH = 7.25) [44–46]. pH values between 7.15 and 7.3 were found in necrotic tumor tissue [47]. As the described variances of pH within tumor tissue do not exceed 1.0 pH-unit [48, 49], we expect the influence of the pH on the NOE-signal *in vivo* to be negligible

### T2 peritumoral edema region

In regard to the peritumoral edema on T2, we found a tendency towards weakly positive correlations of ADC and MTR_asym_ values (0.09<r_Sp_<0.53, p<0.05, n = 9), although 6 patients attenuate this trend displaying negligible (r_Sp_≈0, p<0.05) or insignificant (p>0.05) correlations. Regarding the whole tumor volume, defined as CE-T1 tumor plus peritumoral edema, it has already been stated before that ADC and MTR_asym_ show similar qualitative properties. ADC is lower in the T2 peritumoral edema and increases towards the central necrosis [22], as does the MTR_asym_ signal (always implying that the actual NOE effects behave contrarily to the MTR_asym_ and decrease towards the tumor core) [2, 12, 23]. Therefore, a positive correlation of ADC and MTR_asym_ was to be expected even without knowing the underlying pathophysiology that influences the contrasts’ behavior. In addition, the gradient of both contrasts towards the tumor core might become more eminent in the zone of T2 peritumoral edema as it covers a larger volume and is more homogenous in its structure compared to CE-T1 tumor with its different cell types, vasculogenesis and especially necrotic foci. There have been several studies examining diffusion in peritumoral edema. The overall increased ADC compared to normal appearing white matter is attributed to several factors such as pure vasogenic edema, glial alterations or the breakdown of the extracellular matrix by infiltrating tumor cells. The latter is characteristic for the peritumoral edema of high-grade gliomas [50–53]. Interestingly, Saraswathy et al. [54] found that only the volume of low ADC values throughout the whole tumor (including the T2 peritumoral edema) is predictive of overall survival suggesting that the inclusion of the edematous region is essential for tumor assessment and that an association of ADC with tumor cellularity might only become apparent in this area. The fact that we only observed a weak positive correlation between ADC and MTR_asym_ could be explained by the distinct underlying principles of both contrasts (diffusion of water molecules and protein properties, respectively) which are both linked via cellularity but must have more complex contributions.

### Possible clinical implications and limitations of the study

Generally, there are promising results in regard to the clinical value of NOE mediated imaging. Previously, we were able to show that NOE mediated CEST at 7T provides information on tumor heterogeneity and extent that cannot be obtained from CE-T1 and T2 weighted images. Furthermore, we demonstrated the potential of this endogenous contrast to identify tumor satellites without the use of a contrast agent [23]. The MTR_asym_ maps in this study do confirm these findings descriptively. Furthermore, it is of high interest that NOE mediated CEST provides information on glioblastoma that cannot be acquired with conventional MRI sequences (CE-T1, T2, SWI or DWI). Together with our finding from this study that suggests an association of MTR_asym_ values and tumor cellularity, NOE mediated CEST might be able to contribute to the planning of biopsies, neurosurgical resections or radiation therapy by adding valuable information on tumor infiltration or the identification of most malignant tumor parts.

There are several good arguments to perform CEST-based sequences at 7T: It is known that CEST at 3T suffers from the lower spectral resolution leading to a broadening of both direct water saturation and the CEST effects. Combining this with the lower signal-to-noise-ratio (SNR) makes CEST at lower field strengths very challenging. Per contra, B_1_ and B_0_ field inhomogeneities increase for higher static fields. Therefore, we corrected B_0_ variations as described in Paech et al [23]. Moreover, the absence of a signal variation within the same tissue type from the center of the CEST image to its edges suggests only a small remaining B_1_ influence.

However, there are limitations to this study that need to be discussed:

Firstly, the presented NOE-contrast is based on asymmetry analysis which can be contaminated by other effects such as relaxation changes which were reported to alter the CEST-contrast [39, 55, 56]. Especially T1 can be altered in the area of CE-T1 tumor, which might also explain the large variance of NOE mediated CEST effects here.

Secondly, when calculating the MTR_asym_, there is an interference of several effects such as NOE, APT, direct water magnetization and magnetization transfer of semi-solid tissue components which has to be considered in the evaluation of data. Semi-solid magnetization transfer as a confounder can be neglected in this study as it mainly occurs for higher irradiation powers [4, 57, 58]. The closer to the resonance frequency of water a CEST-effect occurs, the more it is affected by direct water saturation (DS). To correct for DS, the basic and commonly used approach is to subtract the upfield side of the Z-spectrum from the downfield side (yielding MTR_asym_) which was also applied here. However, higher-order correction steps would be needed to obtain a contrast that is exclusively based on the CEST-effect of interest since DS dilutes the CEST-signal and fewer water magnetization is effectively left for the exchange via saturation transfer [27].

Thirdly, a possible inaccuracy lies in the rigid coregistration of the images of sequences with different geometric distortions and might influence both the voxelwise correlation and the correlation with histology and reduce their power. However, the visual inspection of the registration by an experienced neuroradiologist (AR) revealed that EPI distortion artifacts did not lead to a remarkable ROI deviation.

Finally, this is a proof-of-concept study with a relatively small patient collective which has to be borne in mind when interpreting the result. Our findings have to be validated in a larger patient group to determine the contribution of cell density to the NOE-mediated CEST-effects.

In conclusion, NOE-mediated CEST imaging is a new contrast promising insight into pathophysiologic processes in glioblastoma in terms of cell density and protein content. DWI is based on a different methodology as the ADC measures the mean diffusion of water molecules within a defined voxel. However, for the area of T2 peritumoral edema, being less heterogeneous than CE-T1 tumor, our study supposes an association of both contrasts as their underlying principles, protein properties and diffusion of water molecules, are linked via cellularity. Ultimately, within the CE-T1 tumor, NOE mediated CEST based on MTR_asym_ might represent tumor cellularity more accurately than ADC which needs to be verified in future studies with larger patient collectives and histopathological correlation.

## References

[1] DeAngelis LM. Brain Tumors. New England Journal of Medicine. 2001;344(2):114–23. . 11150363 10.1056/NEJM200101113440207

[2] Jones CK, Huang A, Xu J, Edden RAE, Schär M, Hua J, et al Nuclear Overhauser enhancement (NOE) imaging in the human brain at 7T. NeuroImage. 2013;77:114–24. 10.1016/j.neuroimage.2013.03.047 23567889 PMC3848060

[3] Jones CK, Schlosser MJ, van Zijl PC, Pomper MG, Golay X, Zhou J. Amide proton transfer imaging of human brain tumors at 3T. Magnetic resonance in medicine: official journal of the Society of Magnetic Resonance in Medicine / Society of Magnetic Resonance in Medicine. 2006 Sep;56(3):585–92. . 16892186 10.1002/mrm.20989

[4] Zhou J, Lal B, Wilson DA, Laterra J, van Zijl PCM. Amide proton transfer (APT) contrast for imaging of brain tumors. Magnetic Resonance in Medicine. 2003;50(6):1120–6. 14648559 10.1002/mrm.10651

[5] Zhou J, Blakeley JO, Hua J, Kim M, Laterra J, Pomper MG, et al Practical data acquisition method for human brain tumor amide proton transfer (APT) imaging. Magnetic Resonance in Medicine. 2008;60(4):842–9. 10.1002/mrm.21712 18816868 PMC2579754

[6] Sun PZ, Zhou J, Huang J, van Zijl P. Simplified quantitative description of amide proton transfer (APT) imaging during acute ischemia. Magnetic resonance in medicine: official journal of the Society of Magnetic Resonance in Medicine / Society of Magnetic Resonance in Medicine. 2007 Feb;57(2):405–10. . 17260362 10.1002/mrm.21151

[7] Sun PZ, Murata Y, Lu J, Wang X, Lo EH, Sorensen AG. Relaxation-compensated fast multislice amide proton transfer (APT) imaging of acute ischemic stroke. Magnetic resonance in medicine: official journal of the Society of Magnetic Resonance in Medicine / Society of Magnetic Resonance in Medicine. 2008 May;59(5):1175–82. 10.1002/mrm.21591 18429031

[8] Vinogradov E, Sherry AD, Lenkinski RE. CEST: from basic principles to applications, challenges and opportunities. J Magn Reson. 2013 Apr;229:155–72. Pubmed Central PMCID: 3602140. 10.1016/j.jmr.2012.11.024 23273841 PMC3602140

[9] Togao O, Yoshiura T, Keupp J, Hiwatashi A, Yamashita K, Kikuchi K, et al Amide proton transfer imaging of adult diffuse gliomas: correlation with histopathological grades. Neuro-oncology. 2014 Mar;16(3):441–8. Pubmed Central PMCID: 3922507. 10.1093/neuonc/not158 24305718 PMC3922507

[10] Zhou J, Tryggestad E, Wen Z, Lal B, Zhou T, Grossman R, et al Differentiation between glioma and radiation necrosis using molecular magnetic resonance imaging of endogenous proteins and peptides. Nat Med. 2011 Jan;17(1):130–4. Pubmed Central PMCID: 3058561. 10.1038/nm.2268 21170048 PMC3058561

[11] van Zijl PC, Yadav NN. Chemical exchange saturation transfer (CEST): what is in a name and what isn't? Magnetic resonance in medicine: official journal of the Society of Magnetic Resonance in Medicine / Society of Magnetic Resonance in Medicine. 2011 Apr;65(4):927–48. Pubmed Central PMCID: 3148076. 10.1002/mrm.22761 21337419 PMC3148076

[12] Zaiss M, Kunz P, Goerke S, Radbruch A, Bachert P. MR imaging of protein folding in vitro employing nuclear-Overhauser-mediated saturation transfer. NMR in biomedicine. 2013 Dec;26(12):1815–22. 10.1002/nbm.3021 24115020

[13] Vander Heiden MG, Cantley LC, Thompson CB. Understanding the Warburg effect: the metabolic requirements of cell proliferation. Science. 2009 May 22;324(5930):1029–33. Pubmed Central PMCID: 2849637. 10.1126/science.1160809 19460998 PMC2849637

[14] Bentaib A, De Tullio P, Chneiweiss H, Hermans E, Junier MP, Leprince P. Metabolic reprogramming in transformed mouse cortical astrocytes: A proteomic study. Journal of proteomics. 2015 Jan 15;113C:292–314. . 25305589 10.1016/j.jprot.2014.09.019

[15] Iwadate Y, Sakaida T, Hiwasa T, Nagai Y, Ishikura H, Takiguchi M, et al Molecular classification and survival prediction in human gliomas based on proteome analysis. Cancer research. 2004 Apr 1;64(7):2496–501. . 15059904 10.1158/0008-5472.can-03-1254

[16] Ellingson BM, Malkin MG, Rand SD, Connelly JM, Quinsey C, LaViolette PS, et al Validation of functional diffusion maps (fDMs) as a biomarker for human glioma cellularity. Journal of magnetic resonance imaging: JMRI. 2010 Mar;31(3):538–48. Pubmed Central PMCID: 2903058. 10.1002/jmri.22068 20187195 PMC2903058

[17] Sugahara T, Korogi Y, Kochi M, Ikushima I, Shigematu Y, Hirai T, et al Usefulness of diffusion-weighted MRI with echo-planar technique in the evaluation of cellularity in gliomas. Journal of magnetic resonance imaging: JMRI. 1999 Jan;9(1):53–60. . 10030650 10.1002/(sici)1522-2586(199901)9:1<53::aid-jmri7>3.0.co;2-2

[18] Kono K, Inoue Y, Nakayama K, Shakudo M, Morino M, Ohata K, et al The role of diffusion-weighted imaging in patients with brain tumors. AJNR American journal of neuroradiology. 2001 Jun-Jul;22(6):1081–8. . 11415902 PMC7974804

[19] Gupta RK, Cloughesy TF, Sinha U, Garakian J, Lazareff J, Rubino G, et al Relationships between choline magnetic resonance spectroscopy, apparent diffusion coefficient and quantitative histopathology in human glioma. Journal of neuro-oncology. 2000 Dec;50(3):215–26. . 11263501 10.1023/a:1006431120031

[20] Pope WB, Qiao XJ, Kim HJ, Lai A, Nghiemphu P, Xue X, et al Apparent diffusion coefficient histogram analysis stratifies progression-free and overall survival in patients with recurrent GBM treated with bevacizumab: a multi-center study. Journal of neuro-oncology. 2012 Jul;108(3):491–8. Pubmed Central PMCID: 3997502. 10.1007/s11060-012-0847-y 22426926 PMC3997502

[21] Murakami R, Sugahara T, Nakamura H, Hirai T, Kitajima M, Hayashida Y, et al Malignant supratentorial astrocytoma treated with postoperative radiation therapy: prognostic value of pretreatment quantitative diffusion-weighted MR imaging. Radiology. 2007 May;243(2):493–9. . 17356177 10.1148/radiol.2432060450

[22] Crawford FW, Khayal IS, McGue C, Saraswathy S, Pirzkall A, Cha S, et al Relationship of pre-surgery metabolic and physiological MR imaging parameters to survival for patients with untreated GBM. Journal of neuro-oncology. 2009 Feb;91(3):337–51. Pubmed Central PMCID: 3022444. 10.1007/s11060-008-9719-x 19009235 PMC3022444

[23] Paech D ZM, Meissner JE, Windschuh J. Nuclear Overhauser Enhancement mediated Chemical Exchange Saturation Transfer Imaging at 7 Tesla in Glioblastoma Patients. PLoS One. 2014. 10.1371/journal.pone.0104181 PMC4128651 25111650

[24] Schmitt B, Zbýň Š, Stelzeneder D, Jellus V, Paul D, Lauer L, et al Cartilage Quality Assessment by Using Glycosaminoglycan Chemical Exchange Saturation Transfer and 23Na MR Imaging at 7 T. Radiology. 2011 July 1, 2011;260(1):257–64. 10.1148/radiol.11101841 21460030

[25] Zhou J, Hong X, Zhao X, Gao J-H, Yuan J. APT-weighted and NOE-weighted image contrasts in glioma with different RF saturation powers based on magnetization transfer ratio asymmetry analyses. Magnetic Resonance in Medicine. 2013;70(2):320–7. 10.1002/mrm.24784 23661598 PMC3723702

[26] Jin T, Wang P, Zong X, Kim S-G. MR imaging of the amide-proton transfer effect and the pH-insensitive nuclear overhauser effect at 9.4 T. Magnetic Resonance in Medicine. 2013;69(3):760–70. 10.1002/mrm.24315 22577042 PMC3419318

[27] Zaiss M, Bachert P. Chemical exchange saturation transfer (CEST) and MR Z-spectroscopy in vivo: a review of theoretical approaches and methods. Physics in medicine and biology. 2013 Nov 21;58(22):R221–69. . 24201125 10.1088/0031-9155/58/22/R221

[28] Nolden M, Zelzer S, Seitel A, Wald D, Muller M, Franz AM, et al The Medical Imaging Interaction Toolkit: challenges and advances: 10 years of open-source development. International journal of computer assisted radiology and surgery. 2013 Jul;8(4):607–20. 10.1007/s11548-013-0840-8 23588509

[29] Floca R. MatchPoint: On Bridging the Innovation Gap between Algorithmic Research and Clinical Use in Image Registration. In: Dössel O, Schlegel W, editors. World Congress on Medical Physics and Biomedical Engineering, September 7–12, 2009, Munich, Germany. IFMBE Proceedings. 25/4: Springer Berlin Heidelberg; 2010. p. 1105–8.

[30] Greenberg SM, Vernooij MW, Cordonnier C, Viswanathan A, Al-Shahi Salman R, Warach S, et al Cerebral microbleeds: a guide to detection and interpretation. Lancet neurology. 2009 Feb;8(2):165–74. . Pubmed Central PMCID: 3414436. 19161908 10.1016/S1474-4422(09)70013-4 PMC3414436

[31] Radbruch A, Wiestler B, Kramp L, Lutz K, Baumer P, Weiler M, et al Differentiation of glioblastoma and primary CNS lymphomas using susceptibility weighted imaging. European journal of radiology. 2013 Mar;82(3):552–6. 10.1016/j.ejrad.2012.11.002 23238364

[32] Deistung A, Schweser F, Wiestler B, Abello M, Roethke M, Sahm F, et al Quantitative susceptibility mapping differentiates between blood depositions and calcifications in patients with glioblastoma. PloS one. 2013;8(3):e57924 Pubmed Central PMCID: 3605431. 10.1371/journal.pone.0057924 23555565 PMC3605431

[33] Schneider CA, Rasband WS, Eliceiri KW. NIH Image to ImageJ: 25 years of image analysis. Nat Methods. 2012 Jul;9(7):671–5. . 22930834 10.1038/nmeth.2089 PMC5554542

[34] Automatic Nuclei Counter plug-in for ImageJ: Center of Bio-Image Informatics, UC Santa Barbara.

[35] Bonett DG. Sample size requirements for estimating intraclass correlations with desired precision. Statistics in medicine. 2002 May 15;21(9):1331–5. . 12111881 10.1002/sim.1108

[36] Zou KH, Tuncali K, Silverman SG. Correlation and simple linear regression. Radiology. 2003 Jun;227(3):617–22. . 12773666 10.1148/radiol.2273011499

[37] Chen L, Liu M, Bao J, Xia Y, Zhang J, Zhang L, et al The correlation between apparent diffusion coefficient and tumor cellularity in patients: a meta-analysis. PloS one. 2013;8(11):e79008 Pubmed Central PMCID: 3823989. 10.1371/journal.pone.0079008 24244402 PMC3823989

[38] Stadlbauer A, Ganslandt O, Buslei R, Hammen T, Gruber S, Moser E, et al Gliomas: histopathologic evaluation of changes in directionality and magnitude of water diffusion at diffusion-tensor MR imaging. Radiology. 2006 Sep;240(3):803–10. . 16926329 10.1148/radiol.2403050937

[39] Xu J, Zaiss M, Zu Z, Li H, Xie J, Gochberg DF, et al. On the origins of chemical exchange saturation transfer (CEST) contrast in tumors at 9.4 T. NMR in biomedicine. 2014 Jan 29. 24474497. 10.1002/nbm.3075 PMC3972041 24474497

[40] Cramer W. On the biochemical mechanism of growth. The Journal of physiology. 1916 Jul 24;50(5):322–34. . Pubmed Central PMCID: 1420599. 16993346 10.1113/jphysiol.1916.sp001758 PMC1420599

[41] Kalkanis SN, Kast RE, Rosenblum ML, Mikkelsen T, Yurgelevic SM, Nelson KM, et al Raman spectroscopy to distinguish grey matter, necrosis, and glioblastoma multiforme in frozen tissue sections. Journal of neuro-oncology. 2014 Feb;116(3):477–85. 10.1007/s11060-013-1326-9 24390405

[42] Wang M, Kaufman RJ. The impact of the endoplasmic reticulum protein-folding environment on cancer development. Nature reviews Cancer. 2014 Sep;14(9):581–97. 10.1038/nrc3800 25145482

[43] Helmlinger G, Yuan F, Dellian M, Jain RK. Interstitial pH and pO2 gradients in solid tumors in vivo: high-resolution measurements reveal a lack of correlation. Nat Med. 1997 Feb;3(2):177–82. . 9018236 10.1038/nm0297-177

[44] Gerweck LE, Seetharaman K. Cellular pH gradient in tumor versus normal tissue: potential exploitation for the treatment of cancer. Cancer research. 1996 Mar 15;56(6):1194–8. . 8640796

[45] Oberhaensli RD, Hilton-Jones D, Bore PJ, Hands LJ, Rampling RP, Radda GK. Biochemical investigation of human tumours in vivo with phosphorus-31 magnetic resonance spectroscopy. Lancet. 1986 Jul 5;2(8497):8–11. . 2873353 10.1016/s0140-6736(86)92558-4

[46] Hattingen E, Jurcoane A, Bahr O, Rieger J, Magerkurth J, Anti S, et al Bevacizumab impairs oxidative energy metabolism and shows antitumoral effects in recurrent glioblastomas: a 31P/1H MRSI and quantitative magnetic resonance imaging study. Neuro-oncology. 2011 Dec;13(12):1349–63. Pubmed Central PMCID: 3223092. 10.1093/neuonc/nor132 21890539 PMC3223092

[47] Kallinowski F, Vaupel P. pH distributions in spontaneous and isotransplanted rat tumours. British journal of cancer. 1988 Sep;58(3):314–21. . Pubmed Central PMCID: 2246588. 3179183 10.1038/bjc.1988.210 PMC2246588

[48] Vaupel P, Kallinowski F, Okunieff P. Blood Flow, Oxygen and Nutrient Supply, and Metabolic Microenvironment of Human Tumors: A Review. Cancer Research. 1989 December 1, 1989;49(23):6449–65. 2684393

[49] Griffiths JR. Are cancer cells acidic? British journal of cancer. 1991 Sep;64(3):425–7. . Pubmed Central PMCID: 1977628. 1911181 10.1038/bjc.1991.326 PMC1977628

[50] Morita K, Matsuzawa H, Fujii Y, Tanaka R, Kwee IL, Nakada T. Diffusion tensor analysis of peritumoral edema using lambda chart analysis indicative of the heterogeneity of the microstructure within edema. J Neurosurg. 2005 Feb;102(2):336–41. . 15739563 10.3171/jns.2005.102.2.0336

[51] Wang S, Zhou J. Diffusion tensor magnetic resonance imaging of rat glioma models: a correlation study of MR imaging and histology. Journal of computer assisted tomography. 2012 Nov-Dec;36(6):739–44. Pubmed Central PMCID: 3513798. 10.1097/RCT.0b013e3182685436 23192213 PMC3513798

[52] Lu S, Ahn D, Johnson G, Cha S. Peritumoral diffusion tensor imaging of high-grade gliomas and metastatic brain tumors. AJNR American journal of neuroradiology. 2003 May;24(5):937–41. . 12748097 PMC7975803

[53] Engelhorn T, Savaskan NE, Schwarz MA, Kreutzer J, Meyer EP, Hahnen E, et al Cellular characterization of the peritumoral edema zone in malignant brain tumors. Cancer science. 2009 Oct;100(10):1856–62. 10.1111/j.1349-7006.2009.01259.x 19681905 PMC11159753

[54] Saraswathy S, Crawford FW, Lamborn KR, Pirzkall A, Chang S, Cha S, et al Evaluation of MR markers that predict survival in patients with newly diagnosed GBM prior to adjuvant therapy. Journal of neuro-oncology. 2009 Jan;91(1):69–81. Pubmed Central PMCID: 3022437. 10.1007/s11060-008-9685-3 18810326 PMC3022437

[55] Zaiss M, Xu J, Goerke S, Khan IS, Singer RJ, Gore JC, et al Inverse Z-spectrum analysis for spillover-, MT-, and T1- corrected steady-state pulsed CEST-MRI—application to pH-weighted MRI of acute stroke. NMR in biomedicine. 2014 Mar;27(3):240–52. 10.1002/nbm.3054 24395553 PMC4520220

[56] Wu R, Liu CM, Liu PK, Sun PZ. Improved measurement of labile proton concentration-weighted chemical exchange rate (k(ws)) with experimental factor-compensated and T(1) -normalized quantitative chemical exchange saturation transfer (CEST) MRI. Contrast media & molecular imaging. 2012 Jul-Aug;7(4):384–9. . Pubmed Central PMCID: 3415239. 22649044 10.1002/cmmi.505 PMC3415239

[57] Liu D, Zhou J, Xue R, Zuo Z, An J, Wang DJJ. Quantitative characterization of nuclear overhauser enhancement and amide proton transfer effects in the human brain at 7 tesla. Magnetic Resonance in Medicine. 2013;70(4):1070–81. 10.1002/mrm.24560 23238951 PMC3605209

[58] Henkelman RM, Stanisz GJ, Graham SJ. Magnetization transfer in MRI: a review. NMR in biomedicine. 2001;14(2):57–64. 11320533 10.1002/nbm.683

